# Smartphone-Based Image Analysis for Rapid Evaluation of Kiwifruit Quality during Cold Storage

**DOI:** 10.3390/foods11142113

**Published:** 2022-07-15

**Authors:** Hongbo Li, Shuang Lv, Li Feng, Peng Peng, Liangbin Hu, Zhenbin Liu, Subrota Hati, Chitrakar Bimal, Haizhen Mo

**Affiliations:** 1 School of Food and Biological Engineering, Shaanxi University of Science and Technology, Xi’an 710021, China; hongbo715@163.com (H.L.); lvshuang0225@163.com (S.L.); fengli0304@sust.edu.cn (L.F.); hulb@sust.edu.cn (L.H.); zhenbinliu@sust.edu.cn (Z.L.); 2 School of Electrical and Control Engineering, Shaanxi University of Science and Technology, Xi’an 710021, China; pengpeng@sust.eud.cn; 3 SMC College of Dairy Science, Kamdhenu University, Anand 388110, India; subrota_dt@yahoo.com; 4 College of Food Science and Technology, Hebei Agricultural University, Baoding 071001, China; bimalchitrakar@gmail.com

**Keywords:** kiwifruit, quality indexes, rapid evaluation, smartphone-based image analysis, volatile compounds

## Abstract

As a vitamin C–rich fruit, choosing the eating time for kiwifruit with the best quality during the shelf period is still a problem for consumers. This paper mainly focuses on the correlation between cold storage time, quality indexes, volatile flavor compounds of postharvest kiwifruit and RGB value readouts from photos taken by mobile phone. Results indicated that the R to B ratio values (Central R/B) and B to G ratio values (Central B/G) of the central site of kiwifruit were strongly associated with storage time and all quality indicators. The central R/B was negatively correlated with titratable acidity, vitamin C and 2,6-Nonadienal contents and firmness and positively correlated with storage time, weight loss, soluble solids content, total soluble sugars, total plate counts and 1,3-Cyclooctadiene. We provide a novel and smart strategy to predict the shelf life and quality parameters of kiwifruit by capturing and calculating RGB values using a smartphone.

## 1. Introduction

Kiwifruit is a wild deciduous woody vine belonging to the Actinidiaceae family [1]. It is native to southern China and now commercially grown around the world. Currently, the main varieties economically cultivated are the green kiwifruit (*Actinidia deliciosa*) and the sungold kiwifruit (*Actinidia chinensis*). Kiwifruit has been known as “the king of fruit” due to its remarkable abundance of vitamin C, polyphenols, carbohydrates and other health-beneficial components with anti-oxidant and anti-inflammatory properties that can protect against neurological, cardiovascular or cerebrovascular diseases [2]. As one of the elite cultivars of *Actinidia deliciosa* bred in China, the green-fleshed ‘Xuxiang’ kiwifruit is generally harvested in September and October and has several prominent advantages, such as tolerance to storage and excellent mouthfeel.

Kiwifruit belongs to the typical respiratory climacteric type that generates autocatalytic ethylene during ripening [3]. Hence, the fruit is not easy to store at ambient temperature since it readily softens and rots after harvest [4]. Cold or refrigerated storage (0–2 °C) is routinely used to slow down the ripening process and prolong the postharvest life of kiwifruit. Understanding the physiological and biochemical changes during postharvest has important implications to control the pace of senescence and determine the best time for consumption. The postharvest quality changes of kiwifruit are mainly manifested in the firmness, weight, soluble solids content, titratable acidity, total soluble sugars and flavor substances. The quality and flavor during storage are important sensory attributes which not only reflect kiwifruit maturity and aroma characteristics but also affect its consumption. The quality and storability of kiwifruit can be influenced by harvesting time and also temperature. The inter-relationships among quality indexes, flavor compounds and storage time are fundamental for the smart monitoring of kiwifruit quality. Many studies have evaluated the variations in kiwifruit quality and volatile flavor compounds [5], but few have reported the potential correlation of volatile substances and quality indicators with storage time.

Recently, Ma et al. [6] reported an approach to predict the firmness, soluble solids content and pH of kiwifruit based on Vis-NIR spatially resolved spectroscopy. A non-destructive measurement of fruit firmness based on the Sinclair IQ Firmness Tester (SIQ) was proposed to estimate the shelf life of ‘Hayward’ kiwifruit [7]. Moreover, the soluble solids and acidity of kiwifruit can be predicted by chemometrics [8]. Nevertheless, it is still hard to precisely determine the optimal consumption period of this fruit and when decay will occur. A previous study has shown that RGB indices of plum fruit images were correlated with chemical properties of the fruits, so using image processing techniques can evaluate fruit maturity [9]. Here, capturing and analyzing the flesh color of kiwifruit using a smartphone provides a simple and cheap means to predict kiwifruit quality and flavor, which has a wide application prospect for consumers and retailers.

In the present study, we analyzed the correlations between the long-term storage under low temperature (2 °C), volatile compounds and quality indexes based on ‘Xuxiang’ kiwifruit harvested at the end of October. Moreover, to define the shelf life and consumption time, we evaluated the weight loss, firmness, total soluble sugars, soluble solids content, titratable acidity, the total number of colonies, water distribution and volatile compounds. We hope to provide a simple and fast method for ordinary consumers or fruit retailers to quickly evaluate the postharvest quality of kiwifruit.

## 2. Materials and Methods

### 2.1. Plant Materials and Chemicals

300 ‘Xuxiang’ kiwifruit (*Actinidia deliciosa*) with a maturity of about 80% were harvested on 28 October 2021, from a commercial orchard in Mei County, Baoji City, Shaanxi Province, China (latitude 34°17′ N and longitude 107°46′ E). After carefully packing in foam boxes containing ice bags, fruit was transported through 2 °C cold chain truck to the laboratory on the next day. Then, after placing the selected fruit with uniform shape and size into plastic baskets free from mechanical damage, it was wrapped with food preservation film and stored in a refrigerator at 2 °C. Fruit was sampled every 3 d for 1 month. Then, 24 kiwifruits were randomly selected from each time and used to determine changes in physicochemical properties, such as flesh firmness, flesh color, soluble solids content, moisture distribution and flavor substances. Three different kiwifruit were selected for each index test, and each test was repeated in triplicate. All reagents used were of analytical grade.

### 2.2. Analytical Methods

#### 2.2.1. Physical Quality Attributes: Appearance, Fruit Flesh Color, Weight Loss (WL) and Firmness

Photographs were taken every 3 d to observe changes in the appearance and longitudinal section of kiwifruit. Photography was carried out in a small camera box (Shenzhen Zhijie imaging equipment Co., Ltd., Shenzhen, China) with the same lighting conditions (50% brightness, cold white light). With the smartphone readout method, RGB values of different fruit parts (head, central and mesocarp) were captured using the ColorPicker app installed on the mobile phone (Huawei P40, China) [10]. Weights were measured using a Sartorius BSA224S electronic balance (Biobase Biodustry Co., Ltd., Shandong, China) for the same batch of kiwifruit on sampling days. The weight loss (WL) was calculated according to the following formula: WL (%) = (weight of kiwifruit before storage—weight of kiwifruit at sampling day) × 100/weight of kiwifruit before storage. About 1 cm^2^ of the peel on three sides in the equatorial area of each fruit was removed using a peeler. The firmness was measured using a handheld fruit firmness tester (GY-3, Shanghai Grows Precision Instrument Co., Ltd., Shanghai, China) with an 11 mm probe and expressed in N.

#### 2.2.2. Chemical Quality Attributes: Soluble Solids Content (SSC), Total Soluble Sugars (TSS), Titratable Acidity (TA) and Vitamin C (VC)

First, kiwifruit was peeled and homogenized using a home-type blender (SUPOR JR05-300, Zhejiang SUPOR Co., Ltd., Zhejiang, China). The soluble solids content was determined by analyzing the fresh fruit juice with a temperature-compensated refractometer (SATO SK-102R, Jinan Qiandou Industrial Technology Co., Ltd., Shandong, China) and expressed as the degree Brix. The titratable acidity was analyzed according to Dong et al. [11]. Briefly, 10 mL of tenfold dilutions of the kiwifruit juice with two drops of phenolphthalein indicator added was titrated with 0.1 M sodium hydroxide (NaOH) solution using an alkali burette until it turned a light pinkish color. Results are expressed as millimoles of hydronium ions per kg of fresh kiwifruit (mmol kg^−1^). The TSS was measured through the anthrone–sulfuric acid colorimetric method using a spectrophotometer (HIRP V1700G, Hirp International Trade Co., Ltd., Shanghai, China) at 620 nm and calculated with a glucose standard curve, as described by Perveen et al. [12], and are expressed as mg of TSS per L of kiwifruit juice (mg L^−1^). The content of VC was assessed by the titration method using 2,6-dichloroindophenol solution described in the study by Xu et al. [13] and expressed as mg of VC per kg of fresh kiwifruit juice (mg kg^−1^).

#### 2.2.3. Total Plate Counts (TPC)

The total colony numbers of kiwifruit were analyzed according to the method of Song et al. [14]. Samples (20 g) were homogenized and filtered through sterile gauze, then diluted with 0.9% normal saline in three consecutive tenfold dilution series for microbial counts. The diluted kiwifruit juice was plated on the selective agar medium and evenly spread on the surface with a sterile glass rod, then incubated at 37 °C for 48 h. The average value of total plate counts was expressed in CFU g^−1^.

#### 2.2.4. Water Distribution

The moisture distribution of kiwifruit was analyzed by low-field nuclear magnetic resonance (LF-NMR) relaxation measurements performed on a Niumag Benchtop NMR Analyzer PQ001 (Suzhou Niumag Analytical Instrument Co., Ltd., Jiangsu, China) operating at a temperature of 32 °C. Calibration was carried out using free induction decay (FID) sequence. Transverse (T_2_) relaxation times were measured using a Carr–Purcell–Meiboom–Gill (CPMG) pulse sequence. About 2.5 g of sample was tested in each measurement, wrapped in cling film, then packed into a glass tube which was inserted into the NMR analyzer. The CPMG sequence parameters were: 90° and 180° pulse times were set to 7 and 14 µs, respectively; sampling frequency, time waiting (TW), number of echoes (NECH), and number of scans (NS) were 100 kHz, 1000 ms, 18,000 and 14, respectively. Then, the spectrum obtained was inverted, and, according to logarithm coordinates of the raw data and the peak area that corresponded to the water content T_2_, the distribution curves were constructed [15].

#### 2.2.5. Volatile Flavor Compounds (VFC)

The determination of volatile flavor substances in kiwifruit under 2 °C was performed by headspace solid-phase micro-extraction coupled with gas chromatography–mass spectrometry (HS-SPME-GC/MS). For the extraction, a solid-phase microextraction head (50/30 μm DVB/CAR/PDMS, ANPEL Laboratory Technologies Co., Ltd., Shanghai, China) was inserted in sample injection bottles after a 20 min warm-up period and statically adsorbed the volatile flavor compounds for 30 min at 45 °C with continuous stirring with a magnetic stirrer (OLABO HJ-2A, Biobase Biodustry Co., Ltd., Shandong, China). Then, the extraction head was removed and loaded into the GC injection port for desorption at 250 °C for 5 min. The analyses of volatile flavor substances were performed on an Agilent 7890A GC (Agilent Technologies, Inc., Shanghai, China) equipped with a CP-Wax 57 CB column (30 m × 0.25 mm × 0.2 μm) using a 1 mL/min flow rate. The oven temperature was programmed with an initial hold of 5 min at 35 °C, then increased at 5 °C min^−1^ to 150 °C and maintained for 1 min, and finally increased at 7 °C min^−1^ to 250 °C and held for 8 min. The injection volume was set at 1.0 μL with the splitless mode. The MS conditions were as follows: ion source at 230 °C; four-stage bar temperature of 150 °C; ionization voltage of 70 Ev; scan range for full scans of m/z 35–350. Next, the mass spectra and retention times of samples were compared to the NIST/WILEY standard spectral library for compound identification, and area normalization was used to calculate the relative content of components.

### 2.3. Statistical Analysis

OriginPro 8.5 (OriginLab Corp., Northampton, MA, USA) was used for data processing and graphic construction. The z-score normalization method was used to standardize the data. Principal component analysis (PCA) was performed using R language and plotted with the “scatterplot3d” package. Visualization of a correlation matrix in R used the package corrplot.

## 3. Results

### 3.1. Quality Indexes Analysis of ‘Xuxiang’ Kiwifruit

#### 3.1.1. Appearance and Flesh Color

**Figure 1:**
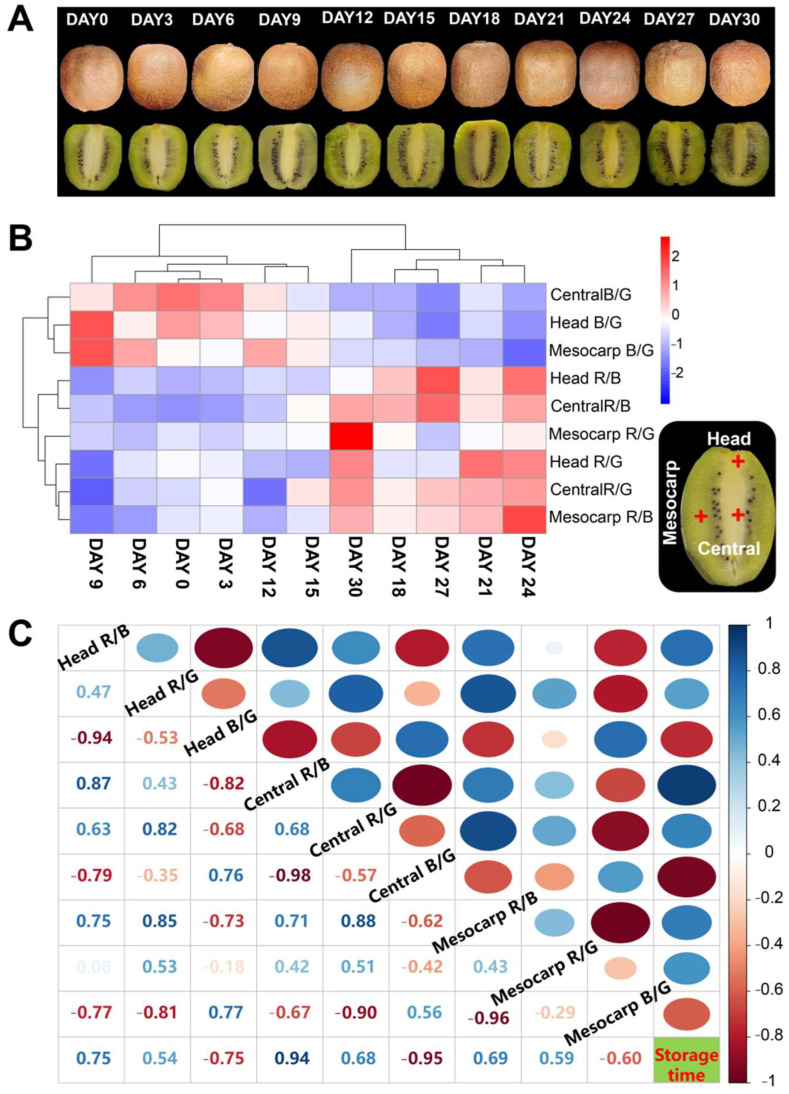
Appearance and flesh color of ‘Xuxiang’ kiwifruit changes during cold storage. Skin and longitudinal section of ‘Xuxiang’ kiwifruit stored at 2 °C for 1 month (**A**). Heatmaps of R/G, R/B and B/G values in different fruit sites (**B**). Correlation between R/G, R/B and B/G values in different fruit sites and storage times (**C**), where +1.0 and −1.0 represent stronger positive and negative correlations between the two features, respectively.

**Figure 2:**
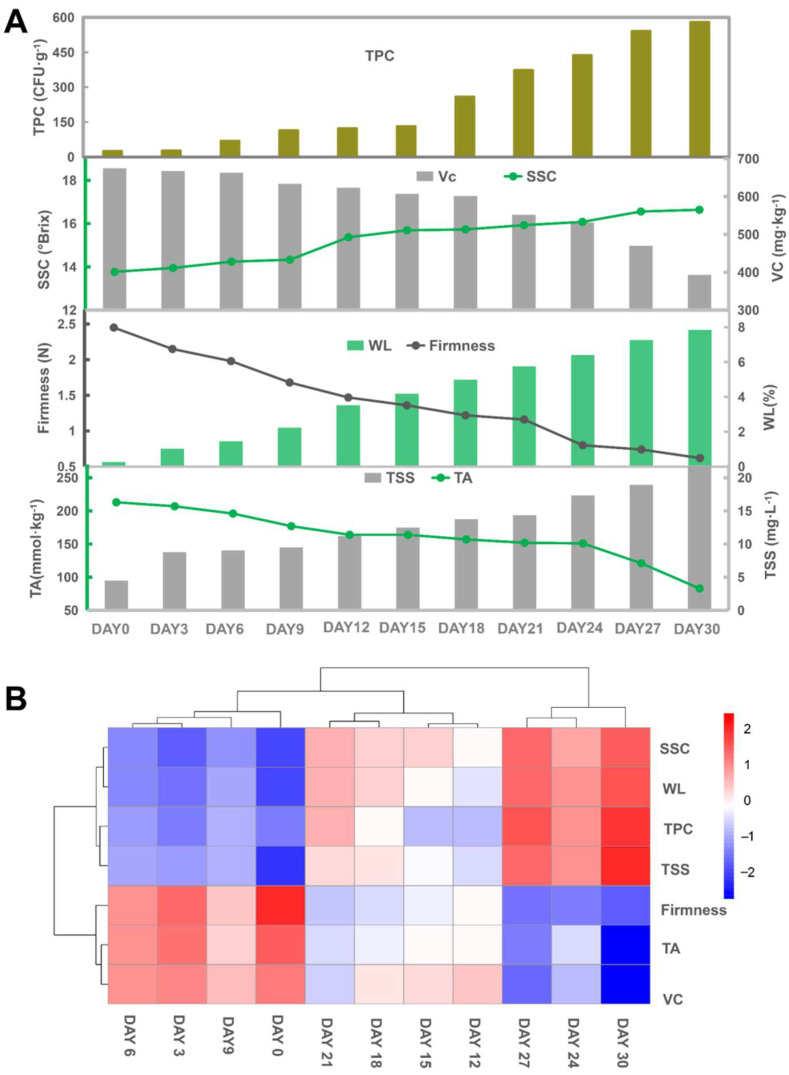
Firmness, weight loss (WL), titratable acidity (TA), total soluble sugars (TSS), total plate count (TPC), soluble solids content (SSC), vitamin C (VC) of ‘Xuxiang’ kiwifruit stored at 2 °C for 1 month (**A**). Heatmaps for quality indicators (**B**).

The changes in skin and longitudinal section of ‘Xuxiang’ kiwifruit during low temperature (2 °C) storage are shown in Figure 1A. The appearance directly affects the merchantability of fruit. Although low-temperature storage can effectively delay the decay of kiwifruit, the changes in appearance and flesh color were not negligible. Each fruit went through the following four phases during storage: softening, peel shrinkage, swelling and decay. The flesh color of kiwifruit gradually turned yellow and the soluble solids content gradually increased with the extension of storage time (Figure 2A), which is consistent with the previous studies that indicated that the fruit flesh color is associated with the content of soluble solids [9,16]. Usually, the higher the content of soluble solids, the darker the yellow flesh.

Changes in R/G (the ratio of R value to G value in the RGB values of kiwifruit flesh), R/B (the ratio of R value to B value) and B/G values (the ratio of B value to G value) in different parts of kiwifruit stored at 2 °C for 1 month are presented in Supplementary Table S1. Heatmaps for R/G, R/B and B/G values in different fruit sites are shown in Figure 1B. After 15 d of postharvest storage, the B/G values in all three parts presented a declining trend, while R/G and R/B values presented an overall increasing trend. The correlation between R/G, R/B and B/G values in different kiwifruit sites and storage times are presented in Figure 1C. The storage time had a significant positive correlation with central R/B (*r* = 0.94) and a significant negative correlation with central B/G (*r* = −0.95). Therefore, the RGB values of the central part can be used to predict the decay time of kiwifruit.

#### 3.1.2. WL, Firmness and Chemical Quality Attributes

At present, some fast and non-destructive methods can be used to predict the SSC, flesh firmness and pH in fruit and vegetables, which has important implications for postharvest quality assessment [6,8,17]. However, simply knowing these parameters is not enough to predict the decay time of kiwifruit. Therefore, the correlations between quality indices and storage time should also be analyzed. Here, the variation of ‘Xuxiang’ kiwifruit quality attributes during storage at 2 °C could be roughly divided into three stages, that is, nearly all characteristics dramatically changed on day 12 and 24 after postharvest storage (Figure 2). 

During postharvest storage, the weight loss of fruit is a normal phenomenon due to water loss and consumption of nutrients in transpiration and respiration, respectively [18,19]. After 1 month of storage at 2 °C, the WL increased linearly from 0.26 to 7.85%. Xu et al. [13] showed that the weight loss of kiwifruit (*Actinidia deliciosa*) displayed a slight sigmoidal shape during the storage period at 4 ± 1 °C with 90–95% RH. Fruit weight loss occurs even at low temperatures. Since senescence and mechanical injuries are directly reflected by softening, firmness is an important physical indicator to evaluate fruit maturity and storability [3]. Thus, the degree of flesh firmness can be used to estimate the remaining shelf life [20]. In the present study, flesh firmness values decreased from 2.45 to 0.62 N as the storage period proceeded. Moreover, the firmness had a positive correlation with TA (*r* = 0.93) and VC (*r* = 0.90) and a negative correlation with WL (*r* = −0.99), SSC (*r* = −0.97), TSS (*r* = −0.97) and TPC (*r* = −0.93). A similar decreasing trend of fruit firmness in ‘Xuxiang’ kiwifruit upon cold storage (0–1 °C) was previously reported by Li et al. [21]. However, in this study, firmness values ranged around 1–15 N during 90 d of storage. We believe that these large differences might be partly due to harvesting ‘Xuxiang’ kiwifruit 1 month later and the 1–2 °C higher storage temperature compared to Li et al. [21]. Indeed, fruit firmness gradually declines as cold storage is prolonged in all cultivars [22]. From another perspective, the content of soluble solids and total soluble sugars presented an increasing trend with the extension of the cold storage period. Li et al. [21] found that the flesh firmness and soluble solids content of ‘Xuxiang’ kiwifruit exhibited downward and upward trends, respectively, during cold storage, consistent with our current results.

The SSC include various water-soluble substances, such as saccharides, vitamins, and some minerals [23]. Here, the SSC in kiwifruit showed an overall rising tendency and ranged between 13.77–16.64°Brix during 1 month of cold storage. Consistent with our results, a rapid increase in SSC was found by Choi et al. [3] and Mitalo et al. [22] within about 25 d of postharvest storage, followed by a leveling-off irrespective of storage temperature or cultivar. Increases in the SSC can occur due to the hydrolysis of starch into soluble sugars [24]. Meanwhile, the content leveled out in the later storage period due to the utilization of sugars produced from starch to supplement the energy consumed by enhanced respiration. Therefore, SSC indicates unique physiological status and is an important indicator for the quality assessment of fruit and vegetables [25,26]. Moreover, a previous study found that an increased rate of soluble solids accumulation was consistent with cool weather in autumn, since low temperatures can lead to starch breakdown [27].

The TA is linked to fruit ripening, nutritional quality and flavor [28,29]. Additionally, maintaining a certain acidity level can improve the flavor of fruit. Overall, the TA of kiwifruit was significantly and inversely correlated with the storage time (Figure 2A). In the ‘Xuxiang’ kiwifruit, the TA declined from 213 to 83 mmol kg^−1^ during 2 °C storage. Additionally, an increased reduction was observed after day 24. At the early harvesting stage, the TA content in all kiwifruit cultivars is high but gradually decreases as the storage time is extended [3,10]. The conversion of organic acids to sugars and their derivatives, or their consumption in respiration, might be the main reasons for the reduced acidity [30]. Additionally, an increased respiration intensity due to decay and softening resulted in the depletion of TA with a concomitant accumulation of TSS. Hence, an optimal sugar/acid ratio with increasing storage time can be determined to provide the best taste.

As an important antioxidant, vitamin C (VC, also known as ascorbic acid) is abundant in kiwifruit [31] and is significant for fruit quality evaluation. Previous studies have indicated that the content of VC in kiwifruit was higher compared to other fruit, including apple and orange [32]. In ‘Xuxiang’ kiwifruit, the content of VC rapidly decreased by approximately 280 mg kg^−1^ FW from day 0 to 30 at 2 °C. Xu et al. [10] reported that the levels of VC in *A. deliciosa* ‘Hayward’ ranged from 102 to 240 mg kg^−1^ FW during storage at 4 ± 1 °C. Krupa et al. [5] reported a higher content of VC, varying from 0.8 to 1.2 g kg^−1^ FW in hardy kiwifruit (*A. arguta* ‘Weiki’) stored at 1 °C for 42 d. Similar changes in VC were observed in our results and the above observations. The similarity is that the content of VC had a downward tendency regardless of cultivars and temperatures, caused by decomposition during postharvest storage.

Soluble sugars are an essential energy source in fruit, also contributing to its taste [33]. Sucrose, glucose and fructose are the major soluble sugars present in all kiwifruit cultivars [34]. In the present study, as the storage period proceed, TSS in ‘Xuxiang’ kiwifruit gradually rose from 4.49 to 21.77 mg L^−1^ FW. In agreement with our results, similar rising trends were reported by Mitalo et al. [35], who also found that total sugars were higher in kiwifruit stored at 22 °C compared to 5 °C. These changes are consistent with the increased expression of genes associated with starch degradation and sugar accumulation in kiwifruit [36,37]. Increasing amounts of TSS might benefit the scavenging of reactive oxygen species (ROS) since many studies have indicated that soluble sugars present some antioxidant capacity [38].

TPC is used to represent the degree of fruit contamination by bacteria, thereby reflecting the fruit health quality. Fruit-based products with a TPC exceeding 1000 CFU g^−1^ are presumed to be inedible. In our study, the TPC in ‘Xuxiang’ kiwifruit increased rapidly after 15 d of storage and reached a peak (580 CFU g^−1^) on day 30. The currently available literature has primarily focused on total bacterial counts in fresh-cut kiwifruit or kiwifruit juice, whereas few studies have targeted TPC changes during storage. Unique to this study, we also found that the TPC showed a positive correlation with WL (r = 0.96), TSS (r = 0.90) and SSC (r = 0.95) and a negative correlation with firmness (r = −0.93), TA (r = −0.92) and VC (r = −0.97).

### 3.2. Moisture Distribution Analysis

**Figure 3:**
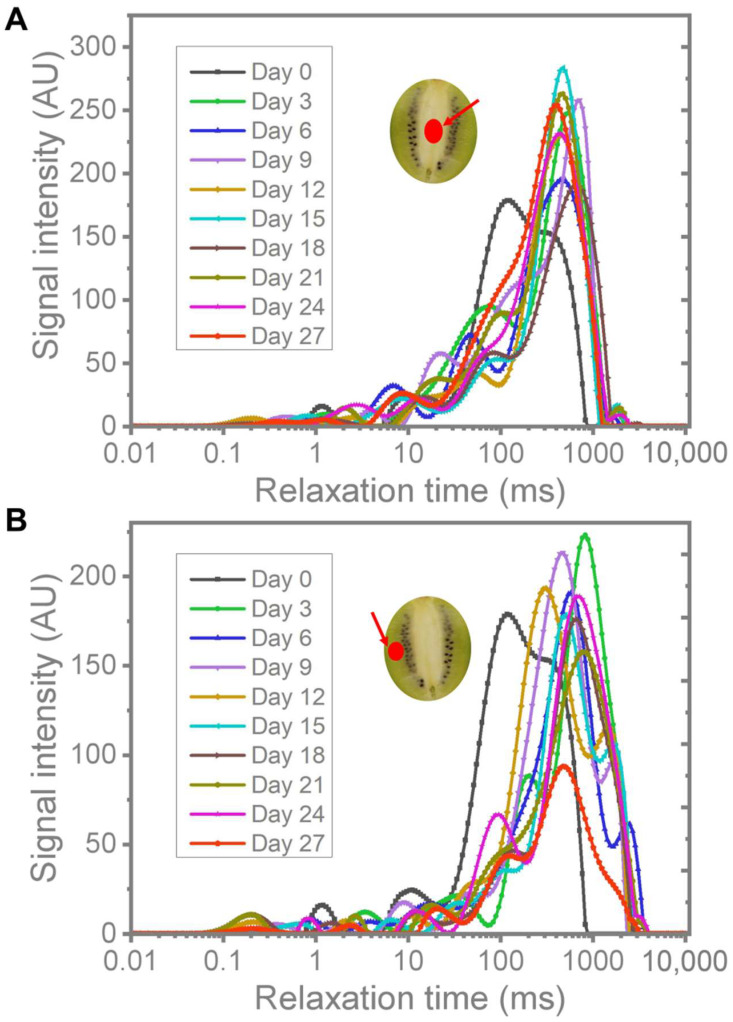
Moisture distribution of kiwifruit in the central part (**A**) and the mesocarp (**B**) during cold storage.

The transverse relaxation time (T_2_) reflects the chemical state of hydrogen protons in the interior of the samples, which is also related to their degree of freedom and tether force [39]. If the T_2_ is large, it indicates that hydrogen protons are bound to a lesser extent to other nonaqueous fractions, and the sample moisture is more likely to be lost. The continuous distribution of T_2_ for different parts of kiwifruit is presented in Figure 3. According to the T_2_, the fitted continuous exponential curves could be roughly divided into three parts: T_21_ (0.01–10 ms), T_22_ (10–100 ms) and T_23_ (100–10,000 ms). The areas of these three components represent the contents of tightly bound, softly bound and free water, respectively [40]. For the central and mesocarp sites of kiwifruit, the relaxation peak of T_23_ on other sampling days shifted to the right compared to day 0, indicating that the water activity increased during postharvest storage at low temperature. However, the relative content of free water increased first, then decreased as the storage time proceeded. This increase in free water might be related to kiwifruit at the ripening stage, whereas the reason for the decrease might be water evaporation of fruit. In the later stages of storage, the fusion of T_22_ and T_23_ peaks was observed, which is derived from severe cell damage leading to water migration from the vacuole to the cytoplasm, finally fusing and avoiding the separation of softly bound and free water [41].

### 3.3. Volatile Flavor Compound Analysis

**Figure 4:**
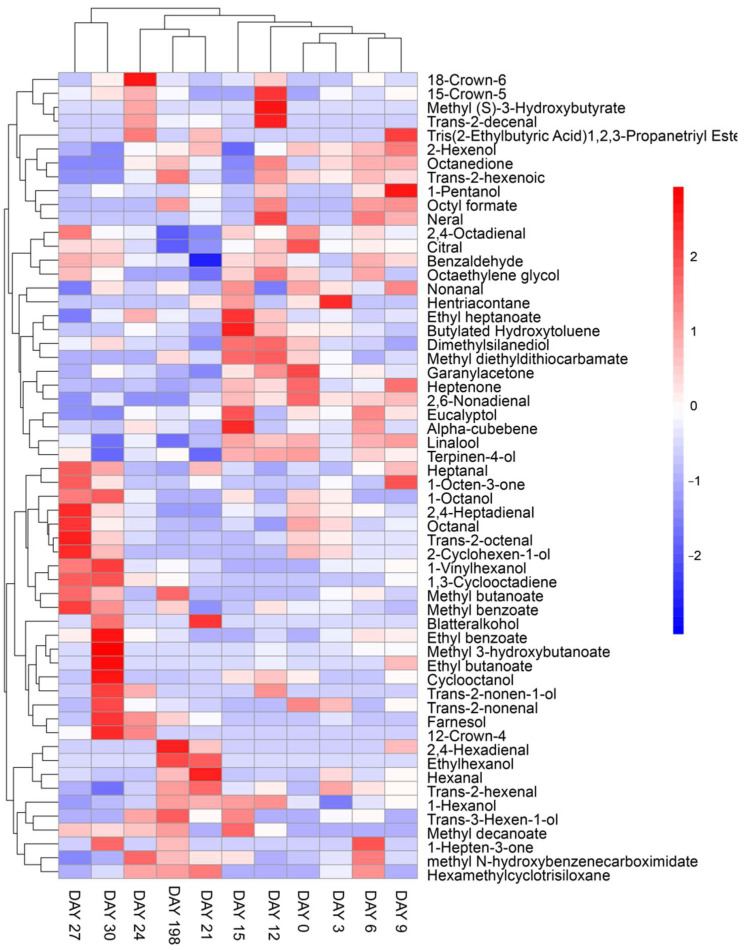
Heatmaps of volatile flavor compounds of ‘Xuxiang’ kiwifruit.

**Figure 5:**
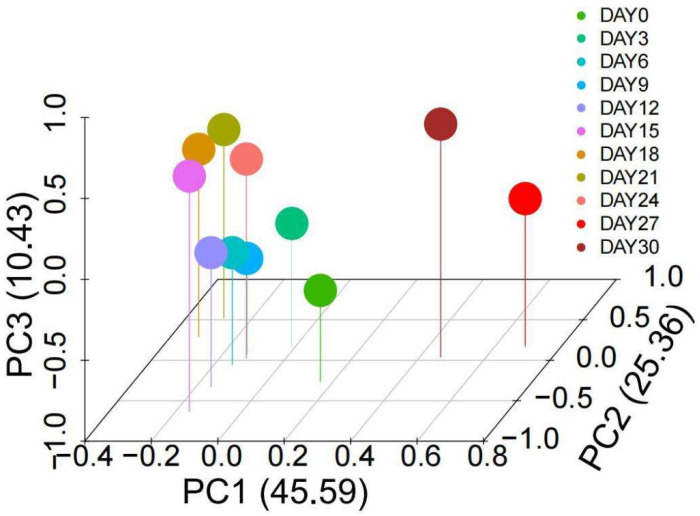
Principal component analysis (PCA) of volatile flavor compounds of ‘Xuxiang’ kiwifruit.

A total of 58 compounds were identified and quantified by HS-SPME-GC/MS in ‘Xuxiang’ kiwifruit during low-temperature storage. These compounds included ketones, alcohols, aldehydes, esters and hydrocarbons as well as a small number of terpenoids (Table 1). The corresponding heatmaps and PCA are shown in Figure 4 and Figure 5, respectively. Volatile compounds of fermented kiwifruit juice and wines made from different cultivars (*Actinidia deliciosa* ‘Xuxiang’ and *Actinidia chinensis* ‘Hongyang’) were identified in previous studies, but very few works have been conducted on the VFC changes of ‘Xuxiang’ kiwifruit during postharvest chilling storage [42].

Aldehydes are essential volatile components of kiwifruit aroma, providing grassy, fresh, and green flavors [43]. In the present study, 44 VFCs were identified at day 0, of which Hexanal and 2-Hexenal, (E)- were the most abundant, corresponding to 16.96 and 31.10%, respectively. Esters are considered positive flavor attributes of kiwifruit aroma, contributing to its fruity and sweet notes. Moreover, ethyl butanoate is a major source of the ripe kiwifruit scent. Here, an overall increase in ester content during storage was detected, reaching a maximum of 31.81% on day 30. Overall, high levels of aldehydes were detected in the early stage of storage, while the content of ester compounds was higher in the mid and late periods. The changes in VFCs were likely caused by gene regulation. The decrease in aldehydes might be related to reduced lipoxygenase (LOX) activity and the increase in esters to the action of alcohol acyltransferases (AATs) during postharvest storage [43]. Additionally, 1-Hexanol, 2-Hexenol and eucalyptol were the major alcohol compounds. Among them, 2-Hexenol, which can be used as a food additive and flavoring, was found at a relatively high level, different from ‘Hongyang’ kiwifruit that presented higher contents of mint-like eucalyptol under natural storage [43]. However, no significant differences were found for the alcohols among different sampling days. Apart from these compounds, heptenone, methyl N-hydroxybenzenecarboximidate and alpha-cubebene were also identified, which might indicate a volatile signature for ‘Xuxiang’ kiwifruit, since they have not been found in other cultivars. We considered that the ‘Xuxiang’ kiwifruit stored for 15 d were at the ripening phase and had the best mouthfeel due to its high content of esters and low content of aldehydes at this stage.

**Figure 6:**
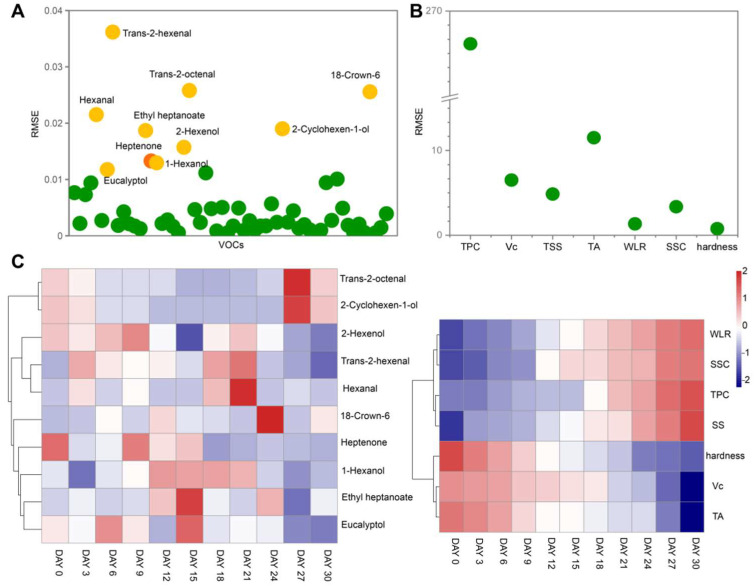
The relations between VFCs—storage time (**A**) and quality indexes—storage time (**B**) were analyzed using the nonlinear least square fit method. Heatmaps analysis of the selected 10 VFCs and all quality indexes with the extension of storage time (**C**).

On day 27, the VFC profile was similar to fruit at day 30 but was significantly different from other sampling days. After 24 d of storage, kiwi changed from the ripening to the decay stage, resulting in elevated levels of compounds related to fruit rot, such as 18-Crown-6, which might account for the above results. The VFCs and storage time were fitted using the nonlinear least-square fit method (Figure 6A). Then, a heatmap with hierarchical clustering between storage time and the 10 VFCs with larger root mean square error (RMSE) was constructed. No significant trends in the contents of the 10 VFCs were detected over time that were different from the quality indexes of the kiwifruit (Figure 6C). Therefore, we further analyzed the correlation between all VFCs identified and storage time. Results indicated that 2,6-Nonadienal (r = −0.78) and 1,3-Cyclooctadiene (r = 0.80) presented a relatively high degree of correlation with storage time (Supplement Figure S1).

### 3.4. Correlations Analysis

**Figure 7:**
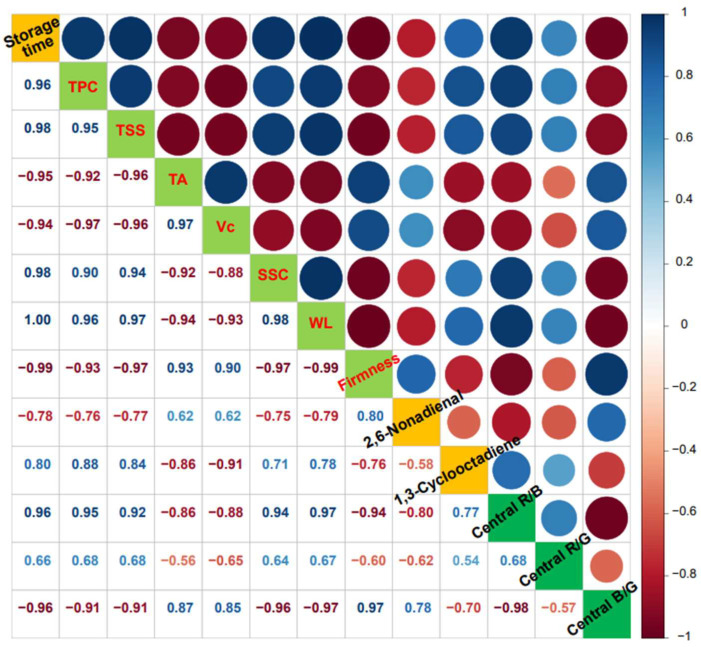
Correlation between all quality parameters, flesh color at the central site of kiwifruit and two VFCs selected with storage time.

The correlation coefficient is a measure of the association between multiple variables. It can be seen from Figure 7 that both central R/B and central B/G of kiwifruit under 2 °C storage presented strong association with storage time and all quality indexes, and two VFCs selected by the above results (Figure 7) exhibited absolute values of correlation coefficients varying between 0.70 and 0.97. It was especially notable that the central R/B was significantly positively correlated with storage time (r = 0.96), TPC (r = 0.95), SSC (r = 0.92), TSS (r = 0.94) and WL (r = 0.97) but negatively correlated with firmness (r = −0.94), which is contrary to central B/G. However, the central R/G revealed a somewhat weaker correlation to all quality indexes and two VFCs. We therefore consider that the flesh color especially the R-to-B ratio values and the B-to-G ratio values of the central part of kiwifruit may be used as an efficient indicator for estimating storage duration, flavor compounds and quality parameters including physical properties, main nutrients and total bacterial count. After estimating the storage time by capturing and computing the RGB values of the kiwifruit using a smartphone, we may further predict the water status at different sites of kiwi at this time.

## 4. Discussion

At present, there exist some non-destructive and intelligent methods to predict the quality changes during storage and remaining shelf life of fruit such as apple, peach and kiwifruit. Already more than ten years ago, it was suggested that the prediction of firmness and soluble solids content would be expected by using multispectral imaging to quantify light backscattering profiles from apple fruit [44]. In recent years, the predictions of soluble solids and titratable acid content in fruit including cherry [45], pear [46] and kiwifruit [6] based on NIR hyperspectral imaging technology have become a hotspot. However, this approach did present some limitations, the most prominent of which is that the hyperspectral imaging system is so expensive that it is associated with great difficulties in practical application by consumers. Additionally, Torkashvand et al. [47] estimated fruit firmness over 6 months using fruit mineral nutrient concentration through multiple linear regressions (MLR) and artificial neural networks (ANNs). A similar method of assessment was reported by Huang et al. [48], who predicted soluble solids content and pH of loquat using fruit mineral elements, but it was not a convenient and speedy method for consumers as a result of requiring the measurement of mineral elements, such as N, P and Ca concentrations. Although a lot of studies on the evaluation of fruit quality have been conducted, there are few studies on predicting quality changes according to the correlation between fruit color and quality indexes. Kaur et al. [9] obtained the mean RGB values of plum fruit through Image Processing Toolbox of MATLAB and established the correlation between color indices and chemical properties so as to quickly estimate the fruit maturity. The present study extracted the RGB values of kiwifruit with mobile phones, which is more suitable for distributors and consumers to rapidly assess the quality and storage time of kiwifruit.

The mobile phone has become one of the essential portable devices in daily life. Overall, the present method based on mobile phone image analysis is more facile and fast compared to other reported prediction methods and provides a new idea for monitoring the postharvest quality of kiwifruit. Moreover, this approach enables kiwifruit growers, greengrocers and consumers to quickly understand the storage time and the best edible period of kiwifruit. At present, there are many image RGB recognition software, which provides the basis for the development of rapid monitoring system based on mobile clients. In future work, we will try to consider more factors which are associated with fruit qualities to establish a more accurate forecasting model so as to further develop the related software for consumers to rapidly determine the remaining shelf life and quality of kiwifruit under 2 °C storage.

## 5. Conclusions

In the present study, the changes in quality indicators and volatile flavor compounds of ‘Xuxiang’ kiwifruit during postharvest low-temperature storage (2 °C) were determined and correlation analyses were performed for each index. We concluded that the firmness, vitamin C and titratable acidity gradually decreased, whereas the weight loss, soluble solids content, total soluble sugars and total plate counts increased with increasing storage time. Results indicated that central R/B and central B/G of kiwifruit were strongly associated with storage time and all quality indicators. The central R/B was negatively correlated with titratable acidity, vitamin C and 2,6-Nonadienal contents and firmness while positively correlated with storage time, weight loss, soluble solids content, total soluble sugars, total plate counts and 1,3-Cyclooctadiene. On this basis, image analysis based on smart phones can reflect the freshness of kiwifruit. We provide a novel and smart strategy to predict shelf life and quality parameters of kiwifruit by capturing and calculating RGB values using a smartphone. Especially for ordinary consumers or fruit retailers at the end of the supply chain, rapid evaluation of the quality of postharvest kiwifruit can avoid missing the best eating period and causing decay and waste.

## Figures and Tables

**Table 1:** Volatile flavor compounds in ‘Xuxiang’ kiwifruit during low-temperature storage at 2 °C.

NO.	RT	Constituents	CAS Number	Formula	MW
1	7.7691	Methyl 3-hydroxybutanoate	1487-49-6	C_5_H_10_O_3_	118.13
2	8.6226	Methyl (S)-3-Hydroxybutyrate	53562-86-0	C_5_H_10_O_3_	118.13
3	8.6646	Methyl butanoate	623-42-7	C_5_H_10_O_2_	102.13
4	9.5601	Ethyl butanoate	105-54-4	C_6_H_12_O_2_	116.16
5	10.2316	Hexanal	66-25-1	C_6_H_12_O	100.16
6	13.1001	Heptanal	111-71-7	C_7_H_14_O	114.19
7	14.0655	Eucalyptol	470-82-6	C_10_H_18_O	154.25
8	14.3593	Trans-2-hexenal	6728-26-3	C_6_H_10_O	98.14
9	15.9543	1-Pentanol	71-41-0	C_5_H_12_O	88.15
10	16.1362	Octanal	124-13-0	C_8_H_16_O	128.21
11	16.5979	1-Octen-3-one	4312-99-6	C_8_H_14_O	126.20
12	16.7098	1-Hepten-3-one	2918-13-0	C_7_H_12_O	112.17
13	17.0457	Octanedione	585-25-1	C_8_H_14_O_2_	142.20
14	17.4235	Ethyl heptanoate	106-30-9	C_9_H_18_O_2_	158.24
15	17.6472	Heptenone	110-93-0	C_8_H_14_O	126.20
16	18.4729	1-Hexanol	111-27-3	C_6_H_14_O	102.17
17	18.7807	Trans-3-Hexen-1-ol	928-97-2	C_6_H_12_O	100.16
18	19.0604	Nonanal	124-19-6	C_9_H_18_O	142.24
19	19.3263	Blatteralkohol	928-96-1	C_6_H_12_O	100.16
20	19.6482	2,4-Hexadienal	142-83-6	C_6_H_8_O	96.13
21	19.8581	2-Hexenol	928-95-0	C_6_H_12_O	100.16
22	20.2498	Trans-2-octenal	2548-87-0	C_8_H_14_O	126.20
23	20.5716	Alpha-cubebene	17699-14-8	C_15_H_24_	204.35
24	20.8514	1-Vinylhexanol	3391-86-4	C_8_H_16_O	128.21
25	21.2572	2,4-Heptadienal	4313-03-5	C_7_H_10_O	110.15
26	22.0127	Ethylhexanol	104-76-7	C_8_H_18_O	130.23
27	22.7964	Benzaldehyde	100-52-7	C_7_H_6_O	106.12
28	22.9643	Trans-2-nonenal	18829-56-6	C_9_H_16_O	140.22
29	23.314	Linalool	78-70-6	C_10_H_18_O	154.25
30	23.7478	Octyl formate	112-32-3	C_9_H_18_O_2_	158.24
31	23.7898	1-Octanol	111-87-5	C_8_H_18_O	130.23
32	24.3355	2,6-Nonadienal	17587-33-6	C_9_H_14_O	138.21
33	24.3914	Methyl decanoate	110-42-9	C_11_H_22_O_2_	186.29
34	24.4754	2,4-Octadienal	30361-28-5	C_8_H_12_O	124.18
35	24.8952	Terpinen-4-ol	562-74-3	C_10_H_18_O	154.25
36	25.0211	Cyclooctanol	696-71-9	C_8_H_16_O	128.21
37	25.119	Methyl benzoate	93-58-3	C_8_H_8_O_2_	136.15
38	25.5527	Trans-2-decenal	3913-81-3	C_10_H_18_O	154.25
39	25.5947	2-Cyclohexen-1-ol	822-67-3	C_6_H_10_O	98.14
40	25.8605	Dimethylsilanediol	1066-42-8	C_2_H_8_O_2_Si	92.17
41	26.0984	Ethyl benzoate	93-89-0	C_9_H_10_O_2_	150.17
42	26.4622	Neral	106-26-3	C_10_H_16_O	152.23
43	26.5182	1,3-Cyclooctadiene	1700-10-3	C_8_H_12_	108.18
44	26.826	Tris(2-Ethylbutyric Acid)1,2,3-Propanetriyl Ester	56554-54-2	C_21_H_38_O_6_	386.52
45	27.0919	Trans-2-nonen-1-ol	31502-14-4	C_9_H_18_O	142.24
46	27.4556	Citral	5392-40-5	C_10_H_16_O	152.23
47	27.8613	Methyl N- hydroxybenzenecarboximidate	1000222-86-6	C_8_H_9_NO_2_	151.16
48	28.6028	Hexamethylcyclotrisiloxane	541-05-9	C_6_H_18_O_3_Si_3_	222.46
49	29.5544	Garanylacetone	3796-70-1	C_13_H_22_O	194.31
50	30.3659	Butylated Hydroxytoluene	128-37-0	C_15_H_24_O	220.35
51	30.7016	Farnesol	4602-84-0	C_15_H_26_O	222.37
52	31.7231	Trans-2-hexenoic	13419-69-7	C_6_H_10_O_2_	114.14
53	31.933	15-Crown-5	33100-27-5	C_10_H_20_O_5_	220.26
54	32.4647	Methyl diethyldithiocarbamate	686-07-7	C_6_H_13_NS_2_	163.30
55	35.0671	18-Crown-6	17455-13-9	C_12_H_24_O_6_	264.32
56	36.0606	12-Crown-4	294-93-9	C_8_H_16_O_4_	176.21
57	36.0746	Octaethylene glycol	5117-19-1	C_16_H_34_O_9_	370.44
58	40.496	Hentriacontane	630-04-6	C_31_H_64_	436.84

Note: RT, retention time; MW, molecular weight; CAS Number, Chemical Abstracts Service Number.

## Data Availability

Data is contained within the article or supplementary material.

## Supplementary Materials

The following supporting information can be downloaded at: https://www.mdpi.com/article/10.3390/foods11142113/s1. Figure S1: Correlation between all quality parameters and VFCs selected with storage time. Table S1: R/G (the ratio of R value to G value in the RGB values of kiwifruit flesh), R/B and B/G values in different parts of kiwifruit stored at 2 °C for 1 month.
