# Supplementary material for: Smartphone-Based Image Analysis for Rapid Evaluation of Kiwifruit Quality during Cold Storage
Source: Foods. 2022 Jul 15;11(14):2113. doi: 10.3390/foods11142113 (PMC9316195; doi:10.3390/foods11142113)
Supplement: Supplementary file 1 [file foods-11-02113-s001.zip › foods-1803445-supplementary.pdf]

Supplemental File

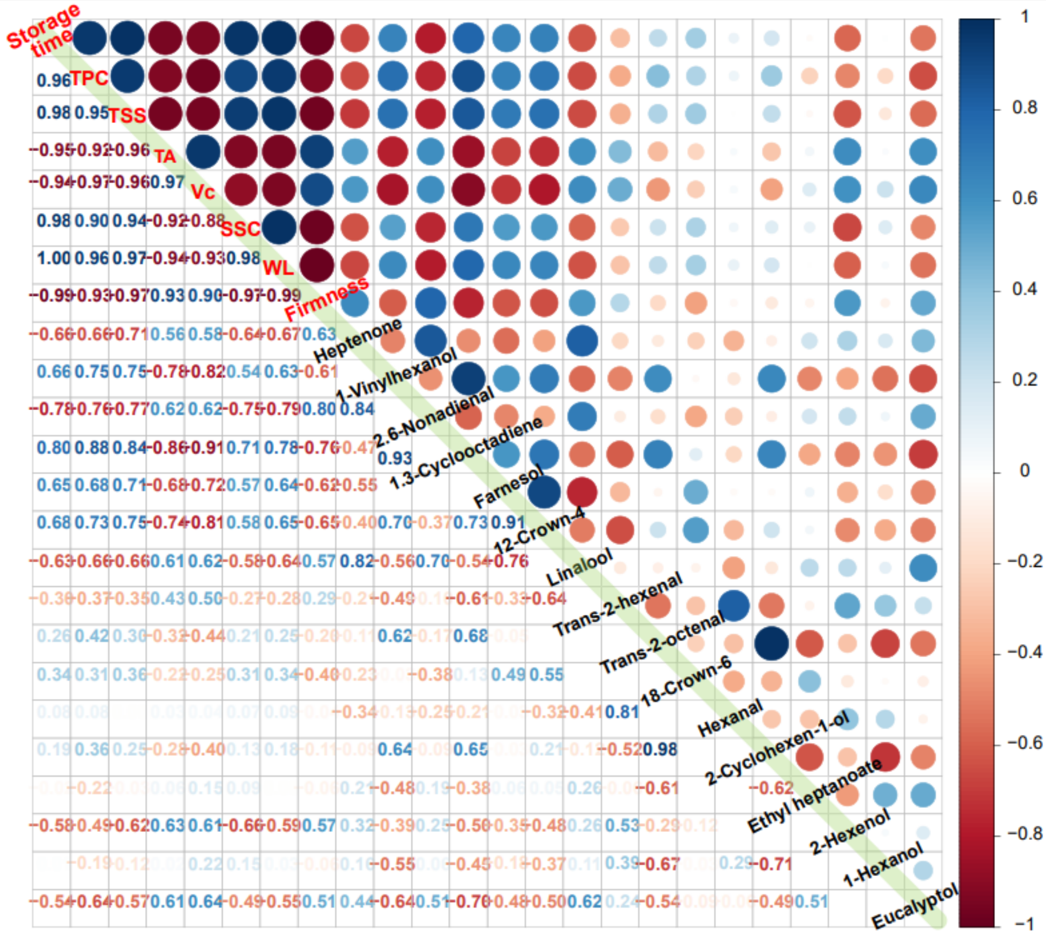

Figure S1. Correlation between all quality parameters and VFCs selected with storage time.

Table S1. R/G (the ratio of R value to G value in the RGB values of kiwifruit flesh), R/B, and B/G values in different parts of kiwifruit stored at 2°C for one month.

	Head R/B	Head R/G	Head B/G	Central R/B	Central R/G	Central B/G	Mesocarp R/B	Mesocarp R/G	Mesocarp B/G
DAY 0	3.21	1.13	0.35	1.64	1.09	0.66	3.21	1.04	0.33
DAY 3	3.46	1.13	0.33	1.71	1.10	0.64	3.29	1.04	0.31
DAY 6	4.10	1.12	0.27	1.72	1.08	0.63	2.73	1.02	0.38
DAY 9	2.46	1.05	0.43	1.95	1.04	0.53	2.43	1.04	0.43
DAY 12	4.38	1.09	0.25	2.00	1.05	0.53	2.81	1.05	0.37
DAY 15	3.96	1.09	0.28	2.37	1.11	0.47	3.19	1.06	0.33
DAY 21	5.64	1.22	0.22	2.46	1.13	0.46	3.91	1.05	0.27
DAY 18	6.57	1.12	0.17	2.79	1.11	0.40	3.56	1.06	0.30
DAY 24	8.40	1.21	0.14	2.89	1.14	0.39	4.79	1.06	0.22
DAY 27	9.18	1.12	0.12	3.26	1.12	0.34	3.71	1.03	0.28
DAY 30	5.03	1.21	0.24	2.86	1.14	0.40	3.96	1.18	0.30
